# Combination of SLC administration and Tregs depletion is an attractive strategy for targeting hepatocellular carcinoma

**DOI:** 10.1186/1476-4598-12-153

**Published:** 2013-12-05

**Authors:** Long Chen, Shuang Zhou, Jie Qin, Heng Hu, Huiying Ma, Binbin Liu, Xuan Wang, Jiaqi Ma, Shenglong Ye, Cuiping Zhong, Guomin Zhou, Chunmin Liang

**Affiliations:** 1 Principle Investigator of the Lab of Tumor Immunology, the Department of Anatomy and Histology & Embryology, Shanghai Medical College, Fudan University, 138 Yixueyuan Road, Shanghai 200032, P R China; 2 Key laboratory of Carcinogenesis and Cancer Invasion, Liver Cancer Institute, Zhongshan Hospital, Shanghai Medical College, Ministry of Education, Fudan University, Shanghai P R China

**Keywords:** SLC, DCs, Tregs, HCC, Anti-tumor immunity

## Abstract

**Background:**

Secondary lymphoid tissue chemokine (SLC) is a key CC chemokine for chemotaxis of immune cells and has been an attractive candidate for anti-tumor treatments. However, among the immune cells recruited by SLC to tumors, the CD25^+^ Foxp3^+^ regulatory T cells (Tregs) compromise the anti-tumor effects. In this study, we proposed the combination therapy of intratumoral co-administration of SLC and anti-CD25 monoclonal antibodies (mAbs). We hypothesized that the intratumoral injections of SLC and depletion of Tregs would have stronger inhibition effects on the progression of hepatocellular carcinoma (HCC) in mice.

**Methods:**

C57BL/6 mice were inoculated subcutaneously with the murine HCC cell line, and mice with visible tumors were treated intratumorally with SLC, SLC plus anti-CD25 mAbs or the control antibodies. The percentages of Tregs, effector CD8^+^ T cells and CD4^+^ T cells were checked in the tumors, lymph nodes, spleen and liver at regular intervals. The levels of intratumoral IL-12, IFN-γ, IL-10 and TGF-β1 were evaluated. The final anti-tumor effects were measured by the tumor volume and weight as well as the intratumoral activity of MMP2 and MMP9. Bone-marrow-derived dendritic cells were used to explore the mechanisms of maturation induced by SLC in vitro.

**Results:**

Our experiments showed the combination therapy significantly decreased the frequency of Tregs, and increased CD8^+^ T cells and CD4^+^ T cells at tumor sites. These alterations were accompanied by an increased level of IL-12 and IFN-γ, and decreased level of IL-10 and TGF-β1. Unexpectedly, we observed a significantly decreased percentage of Tregs, and increased CD8^+^ T cells and CD4^+^ T cells in the lymph nodes, spleen and liver after the combination therapy. The growth and invasiveness of HCC was also maximally inhibited in the combination therapy compared with the SLC alone. Furthermore, we confirmed SLC induced the maturation of DCs via NF-κB p65 and this maturation would benefit the combination therapy.

**Conclusions:**

Our data demonstrated that intratumoral co-administration of SLC and anti-CD25 mAbs was an effective treatment for HCC, which was correlated with the altered tumor microenvironment and systemically optimized percentages of Tregs, CD8^+^ T cells and CD4^+^ T cells in peripheral immune organs.

## Background

Secondary lymphoid tissue chemokine (SLC, also known as CCL21) is an important CC chemokine which can recruit various immune cells such as dendritic cells (DCs) and T cells [1]. In addition, recent research from our group and others has demonstrated SLC can induce the maturation of DCs [2,3]. These properties make SLC an attractive candidate for anti-tumor treatments [4]. Indeed, several studies have demonstrated intratumoral administration of SLC elicits significant tumor regression [5,6]. We have also shown that intratumorally up-regulating the level of SLC in hepatocellular carcinoma (HCC) is an effective strategy to halt the progression of tumors [3,7].

Among the immune cells at tumor sites, there exists a group of regulatory T cells (Tregs) that are key components in tumor immune suppression. They are broadly identified as CD4^+^ T cells that highly express CD25 and Foxp3 [8]. Tregs act by suppressing the activation, proliferation and function of other immune cells [9]. Increasing evidence indicates that Tregs accumulate in the tumor microenvironment (TME) and consequently inhibit the anti-tumor immunity [10-12]. This could explain the efficacy of anti-CD25 monoclonal antibodies (mAbs) treatment in inducing tumor rejection in animal models [13-15].

In light of these results, we proposed the combination therapy of intratumoral co-administration of SLC and anti-CD25 mAbs. In this study, we found the combination therapy decreased the frequency of Tregs, and increased CD8^+^ T cells and CD4^+^ T cells at tumor sites, with increased levels of IL-12 and IFN-γ and decreased IL-10 and TGF-β1. Importantly, we observed the systemic optimization of Tregs, CD8^+^ T cells and CD4^+^ T cells in the lymph nodes, spleen and liver after the combination therapy. The growth and invasiveness of HCC was maximally inhibited in the combination therapy. *In vitro* experiments confirmed that SLC-induced maturation of DCs was mediated by the NF-κB p65, which benefited the SLC-based combination therapy. These data provided interesting clues for clinical immunotherapy in HCC.

## Results

### Depletion of Tregs by anti-CD25 mAbs in the murine HCC model

**Figure 1:**
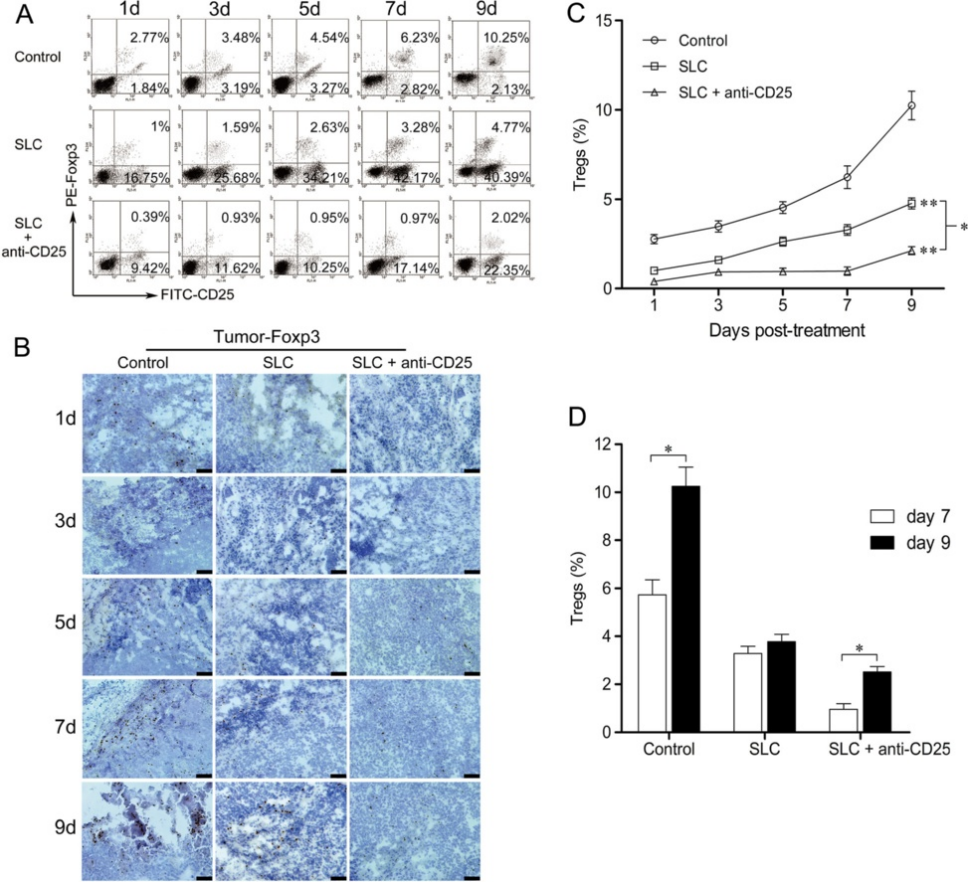
**Depletion of Tregs by anti-CD25 mAbs in SLC-treated mice. (A)** Representative FACs plots of CD25^+^ Foxp3^+^ Tregs gated on CD4^+^ T cells in tumors on day 1 to 9 post-treatment. **(B)** Representative staining of Foxp3^+^ Tregs in the tumor tissue (dark brown). **(C)** A curve diagram was summarized for the results from all treated and control mice. **(D)** The sharp increased frequency of Tregs from day 7 to day 9. Control: control group; SLC: SLC group; SLC + anti-CD25: combination therapy group. Values were presented as mean ± SD; n = 5 mice/group. * *P* < 0.05, ** *P* < 0.01 *vs* control or indicated comparison on day 1 to 9. Results were obtained in three independent experiments. Bar = 50 μm.

First we verified the efficacy of depletion of Tregs after anti-CD25 mAbs injection in tumors. Representative data of CD25^+^ Foxp3^+^ Tregs was shown by FACs and IHC (Figure 1A and B). The results from all treated and control mice were summarized and provided in a curve diagram. As shown in Figure 1C, from day 1 to 9 post-treatment, intratumoral Tregs remained essentially constant at the significantly lowest level in SLC-anti-CD25 mAbs treated mice, whereas Tregs in the control mice were the highest and showed a linear increase. The SLC group showed a modest increase of Tregs with a similar pattern as the control group. Interestingly, there was a sharp increase of Tregs in all three groups from day 7 to day 9 (Figure 1C and D).

### Dynamic changes of CCR7 and Foxp3 in tumors under the combination therapy

**Figure 2:**
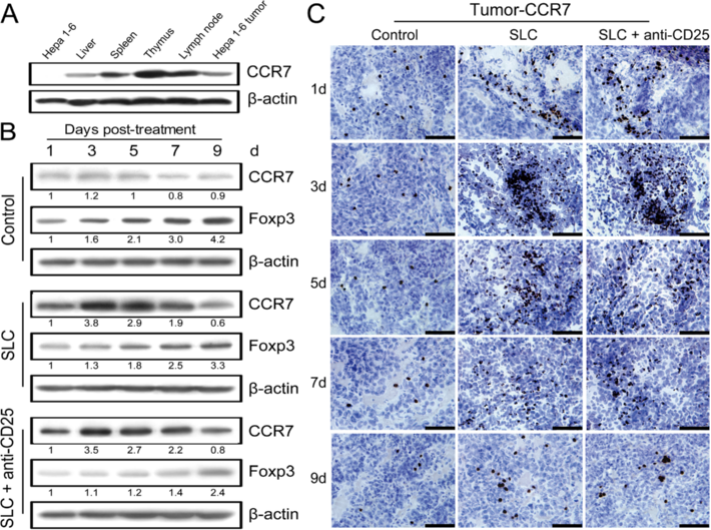
**Dynamic changes of CCR7 and Foxp3 in tumors after the treatments. (A)** Expression of CCR7 in Hepa 1–6 cells, tumors and immune organs. **(B)** Expression of CCR7 and Foxp3 in tumors on day 1 to 9 post-treatment. **(C)** Representative staining of CCR7 (dark brown) in tumors on day 1 to 9 post-treatment. Control: control group; SLC: SLC group; SLC + anti-CD25: combination therapy group. β-actin was used as a quantitative control. Densitometer quantitation was relative to the first data set in each case (indicated by a value of 1). All data are representative of at least two independent experiments. Bar = 50 μm.

CCR7 was detected in the thymus, lymph nodes, spleen, liver and Hepa 1–6 tumor, but not in the Hepa 1–6 cell line (Figure 2A). Therefore, the chemotactic effect of SLC can be measured by the level of intratumoral CCR7, and depletion of Tregs can be defined by the level of Foxp3. We observed that the combination therapy group and SLC group showed steady up-regulation of CCR7 on day 1 to 7 post-treatment but with the obvious down-regulation on day 9, whereas the control group remained the basal level of CCR7 during the same period (Figure 2B and C). In contrast, we noticed the combination therapy group showed the lowest level of Foxp3 at each time point while all 3 groups exhibited a gradually increased level of Foxp3 on day 1 to 9 post-treatment (Figure 2B).

### Altered frequency of CD4^+^ and CD8^+^ T cells at tumor sites

**Figure 3:**
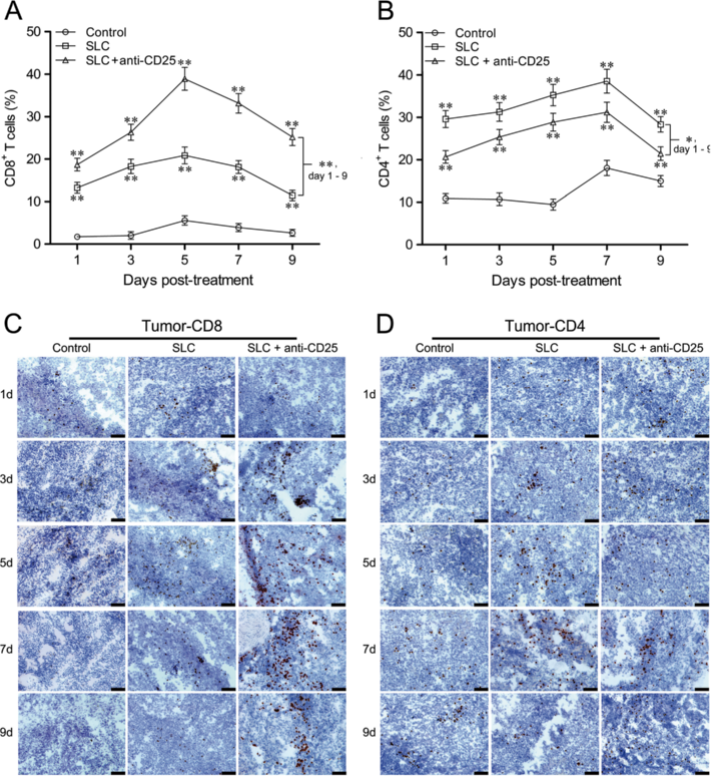
**Frequency of CD8**^**+ **^**T cells and CD4**^**+ **^**T cells in tumors after the treatments.** Frequency of CD8^+^ T cells **(A)** and CD4^+^ T cells **(B)** in tumors was analyzed on day 1 to 9 post-treatment by FACs gated on lymphocytes. Representative staining of CD8^+^ T cells **(C)** and CD4^+^ T cells **(D)** in tumors (dark brown) was shown by IHC. Control: control group; SLC: SLC group; SLC + anti-CD25: combination therapy group. Values were presented as mean ± SD; n = 5 mice/group. * *P* < 0.05, ** *P* < 0.01 *vs* control or indicated comparison on day 1 to 9. Results were obtained in three independent experiments. Bar = 50 μm.

After quantifying infiltrating T cells in tumors, we found the combination therapy group had the highest level of CD8^+^ T cells (*P* < 0.01) on day 1 to 9, while the control group showed the lowest level (*P* < 0.01) (Figure 3A and C). On the contrary, the SLC group manifested the highest level of CD4^+^ T cells (*P* < 0.05 and *P* < 0.01) and the combination therapy group showed a modest increase (*P* < 0.01) (Figure 3B and D) on day 1 to 9.

### Cytokines profiles of IL-12, IFN-γ, IL-10 and TGF-β1 were skewed in the TME

**Figure 4:**
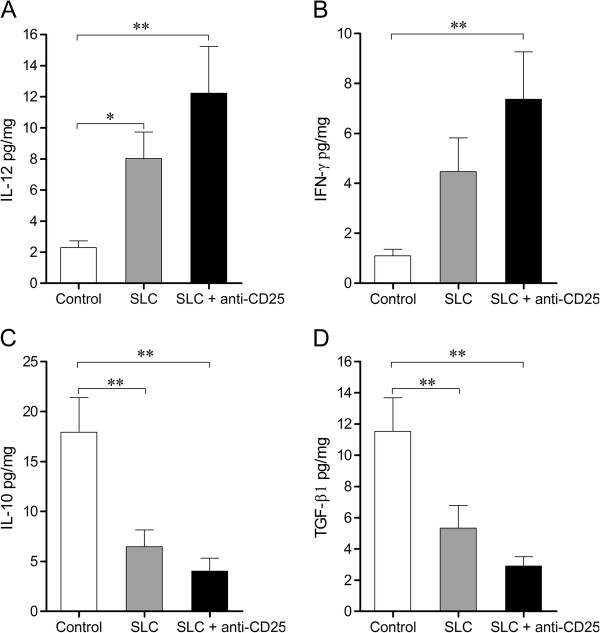
**Levels of the four cytokines in the tumor microenvironment.** The tumor samples on day 5 post-treatment were harvested and intratumoral levels of IL-12 **(A)**, IFN-γ **(B)**, IL-10 **(C)** and TGF-β1 **(D)** were evaluated by ELISA. Control: control group; SLC: SLC group; SLC + anti-CD25: combination therapy group. Values were presented as mean ± SD; n = 5 mice/group. * *P* < 0.05, ** *P* < 0.01 for indicated comparison. Results were obtained in three independent experiments.

Compared with the control group, both treated groups showed significantly higher levels of IL-12 (Figure 4A) and IFN-γ (Figure 4B), but significantly lower levels of immunosuppressive mediators IL-10 (Figure 4C) and TGF-β1 (Figure 4D) on day 5 post-treatment, a representative time-point for Tregs depletion and tumor growth inhibition. However, the levels of the four cytokines were not significantly different between the SLC group and the combination therapy group.

### Systemic optimization of Tregs, CD8^+^ and CD4^+^ T cells in the lymph nodes, spleen and liver

**Figure 5:**
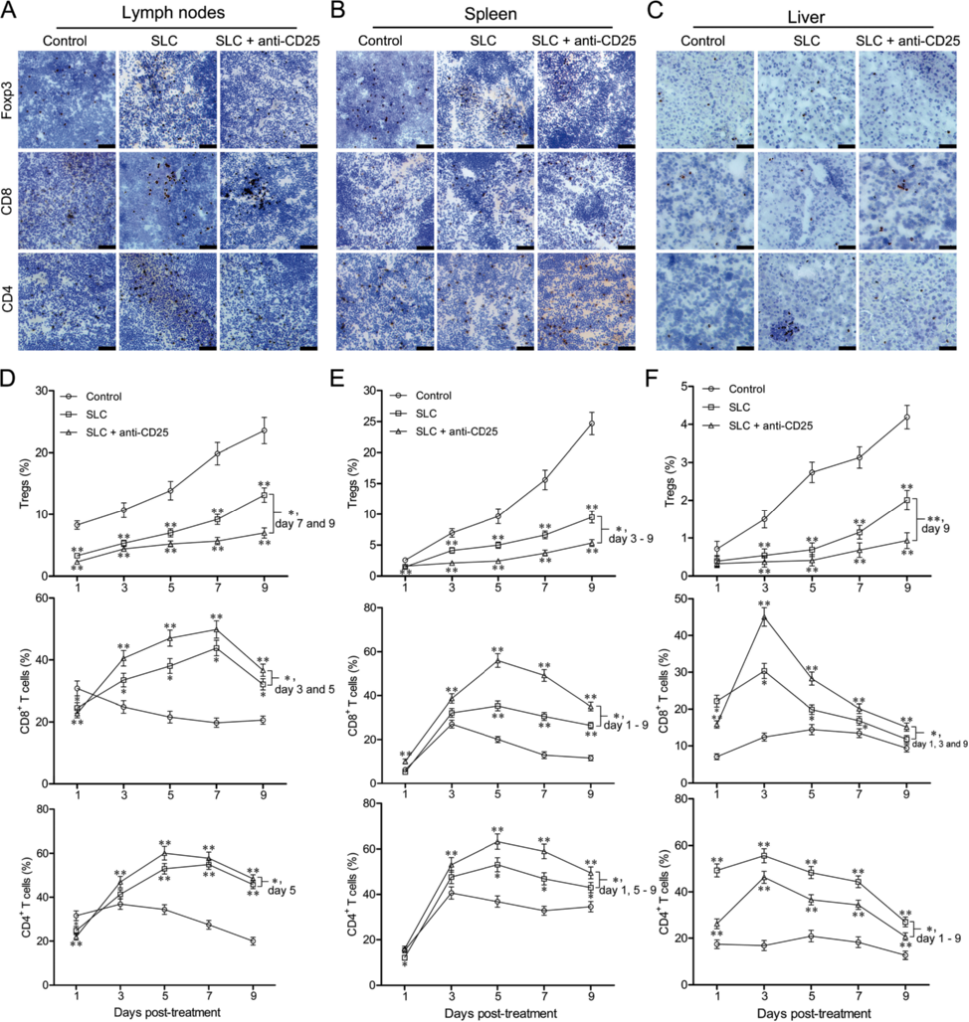
**Optimization of Tregs, CD8**^**+ **^**and CD4**^**+ **^**T cells in the lymph nodes, spleen and liver.** Representative staining of Foxp3^+^ Tregs, CD8^+^ T cells and CD4^+^ T cells was shown by IHC in the lymph nodes **(A)**, spleen **(B)** and liver **(C)** on day 5 post treatment (dark brown). Quantitative analysis of Foxp3^+^ Tregs, CD8^+^ T cells and CD4^+^ T cells was performed by FACs in the lymph nodes **(D)**, spleen **(E)** and liver **(F)** on day 1 to 9 post-treatment. Control: control group; SLC: SLC group; SLC + anti-CD25: combination therapy group. Values were presented as mean ± SD; n = 5 mice/group. * *P* < 0.05, ** *P* < 0.01 *vs* control or indicated comparison on day 1 to 9. Results were obtained in three independent experiments. Bar = 50 μm.

We next evaluated the frequency of Tregs, CD8^+^ and CD4^+^ T cells in peripheral immune organs as well as in the liver. In lymph nodes, IHC indicated decreased Tregs and increased CD8^+^ and CD4^+^ T cells in the two treated groups on day 5 post-treatment (Figure 5A). FACs results showed the frequency of Tregs in the two treated groups was lower than the control group (*P* < 0.01) and specifically the combination therapy group had a lower frequency of Tregs on day 7 and 9 compared with the SLC group (*P* < 0.05) (Figure 5D). The two treated groups had higher levels of CD8^+^ and CD4^+^ T cells compared with the control on day 3 to 9 (*P* < 0.01); between the two treated groups, the combination therapy group had a significantly higher level of CD8^+^ T cells on day 3 and 5, and a significantly higher level of CD4^+^ T cells on day 5 (Figure 5D). The cryostat sections of the spleen on day 5 post-treatment by IHC showed decreased Tregs and increased CD8^+^ and CD4^+^ T cells in combination therapy group and SLC group (Figure 5B). Quantitative results confirmed that the two treated groups had a decreased percentage of Tregs and increased percentages of CD8^+^ and CD4^+^ T cells compared with the control; furthermore, the combination therapy group had the significantly lowest level of Tregs (day 3 to 9), the highest level of CD8^+^ (day 1 to 9) and CD4^+^ T cells (day 1 and 5 to 9) (Figure 5E). The two treated groups showed a similar pattern of Tregs, CD8^+^ and CD4^+^ T cells in the liver: decreased frequency of Tregs and increased frequency of CD8^+^ and CD4^+^ T cells; however, the combination therapy group had a lower level of Tregs only on day 9 and the higher level of CD8^+^ T cells on day 3 and 9, compared with SLC group (Figure 5C and F). SLC group had the highest level of CD4^+^ T cells on day 1 to 9 (*P* < 0.05) (Figure 5F).

### The inhibition of the established HCC was stronger under the combination therapy

**Figure 6:**
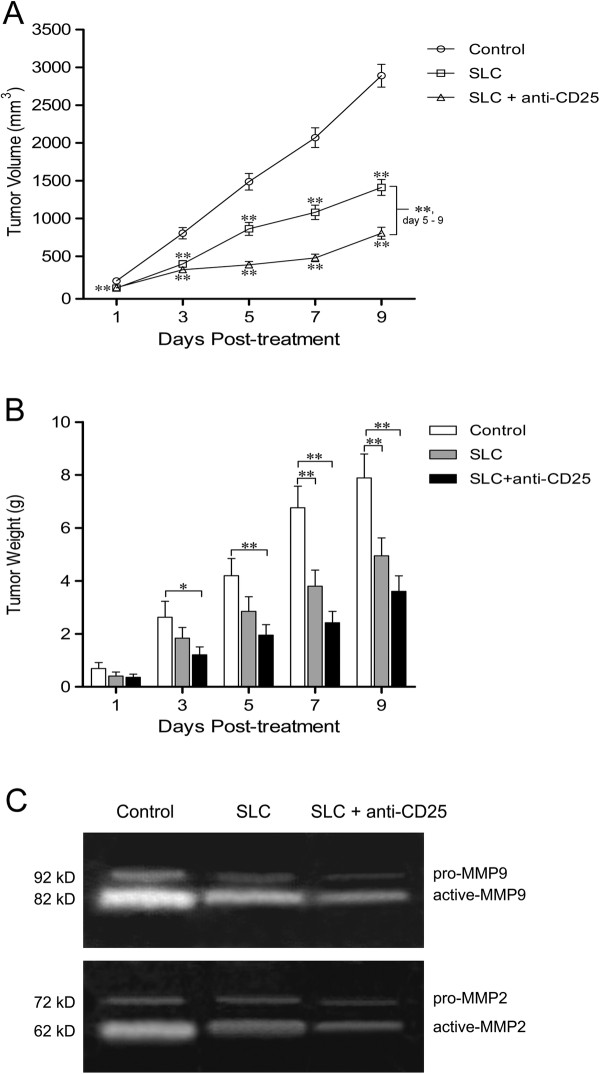
**Maximal inhibition of the established HCC under the combination therapy.** Tumor size was monitored on day 1 to 9 post-treatment **(A)** and mice were sacrificed and the entire tumor was weighed **(B)**. Gelatin zymography was used to detect intratumoral MMP-2 and MMP-9 activity **(C)**. Control: control group; SLC: SLC group; SLC + anti-CD25: combination therapy group. Values were presented as mean ± SD; n = 5 mice/group. * *P* < 0.05, ** *P* < 0.01 *vs* control or indicated comparison on day 1 to 9. Results were obtained in three independent experiments;

The anti-tumor effects of the combination treatment were assessed by monitoring the tumor volume and weight. We found that the combination therapy caused maximal inhibition in HCC volume (*P* < 0.01, day 5 to 9) (Figure 6A). Along with the reduction of volume, the combination treatment also significantly reduced tumor weight (Figure 6B). Analyzing the invasiveness of tumors by gelatin zymography, we found both pro- and active forms of MMP-2 and MMP-9 were reduced in tumors treated by the combination therapy (Figure 6C) on day 5 post-treatment.

### SLC induced the maturation of DCs via the NF-κB p65 in vitro

**Figure 7:**
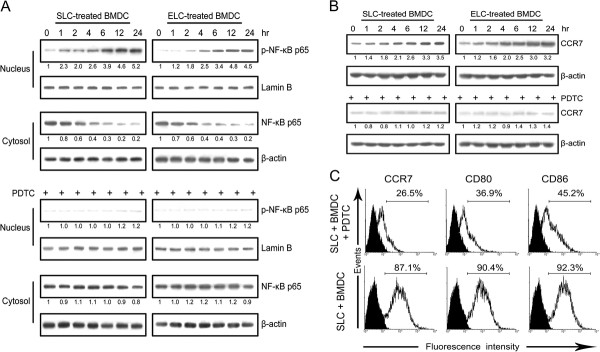
**NF-κB p65 mediated the maturation of DCs downstream of SLC.** BMDCs were pre-treated with PDTC for 1 h and then cultured with SLC or ELC for 1, 2, 4, 6, 12, 24 h. Whole cell lysates, nuclear lysates and cytosol lysates were analyzed for NF-κB p65 and phosphorylated NF-κB p65 **(A)**, and CCR7 **(B)**. BMDCs were pre-treated with PDTC for 1 h and then cultured with SLC for 24 h. Expression of CCR7, CD80 and CD86 was analyzed by FACs **(C)**. Densitometer quantitation was relative to the first data set in each case (indicated by a value of 1).

It has been demonstrated that the anti-tumor effects of SLC can be partially attributed to the maturation of DCs induced by SLC in tumors [6,16]; however, it is still not clear about the detailed mechanisms underlying the SLC-induced maturation of DCs. We analyzed the expression profiles of bone-marrow-derived dendritic cells (BMDCs) after stimulation with SLC by the genes array. The array data showed that various genes were up-regulated after the stimulation, such as the PKC pathway (Ca^2+^, MEK, etc.), PI-3 kinase pathway and NF-κB pathway (Table 1). We focused on the NF-κB pathway in the present study, which was important in the maturation of DCs. We found both SLC and ELC stimulation elicited up-regulation of phosphorylated NF-κB p65 (p-NF-κB p65) and down-regulation of NF-κB p65, and these changes were blocked by PDTC, the specific inhibitor of NF-κB activation (Figure 7A), which was consistent with the array data. Phenotypes assessment suggested that during this maturation BMDCs up-regulated CCR7, CD80 and CD86, while PDTC significantly inhibited this process (Figure 7B and C).

**Table 1:** Up-regulated genes in BMDCs treated with SLC for 1 or 12 hours

**Unigene**	**Genebank**	**Description**	**Gene name**	**Exp.1**	**Exp.2**	**Exp.2/Exp.1**
*Ca*^*2+ *^*pathway*						
Mm.3064	NM_007922	M.musculus mRNA for elk1 protein	Elk-1	1.509E-01	3.941E-01	2.612E + 00
Mm.195050	NM_007923	ELK4, member of ETS oncogene family	Sap 1a	2.183E-02	1.673E-01	7.662E + 00
*PKC pathway*						
Mm.896	NM_008362	Interleukin 1 receptor, type I	IL-1R1	1.194E-04	9.819E-04	8.223E + 00
*Protein tyrosine kinase pathway*						
Mm.22288	NM_007631	Cyclin D1	Cyclin D1	2.426E-02	3.758E-01	1.549E + 01
Mm.16110	NM_007633	Cyclin E1	Ccnel	4.159E-02	2.403E-01	5.778E + 00
Mm.35867	NM_009830	Cyclin E2	Cyclin E2	2.048E-01	4.335E-01	2.117E + 00
*NF-*κ*B pathway*						
Mm.76649	NM_011693	Vascular cell adhesion molecule 1	VCAM-1	9.880E-02	2.347E-01	2.376E + 00
*JAK-STAT pathway*						
Mm.34446	NM_007669	Cyclin-dependent kinase inhibitor 1A (P21)	p21Waf1/p21cip	8.938E-03	7.725E-02	8.642E + 00

Exp.1 represented the expression of the genes in BMDCs treated with SLC for 1 hour; Exp.2 represented the expression of genes in BMDCs treated with SLC for 12 hours; Exp.2/Exp.1 meant the fold change.

## Discussion

In this study we demonstrated that intratumoral injection of SLC plus anti-CD25 mAbs maximally inhibited tumor progression in a murine HCC model. To our knowledge, this was the first report to demonstrate the remarkable effects of SLC-based combination therapy in HCC.

We found that the frequency of Tregs remained the lowest in the combination therapy group. This result was consistent with previous studies identifying the efficacy in depleting Tregs by anti-CD25 mAbs treatment [13,17,18]. As mature DCs mainly activate immune response in contrast to immature DCs which easily induce Tregs, we propose the maturation of DCs induced by SLC also contributed to the decreased Tregs. The intratumoral level of Foxp3 was reversely correlated with tumor progression [19,20] and we found the level of Foxp3 in the combination therapy remained the lowest among the 3 groups, which represented a beneficial anti-tumor effect.

At the same time, we found the increased level of CCR7 at tumor sites in both the SLC group and combination therapy group, concordant with the chemotactic properties of SLC. We also noticed a sharp drop of CCR7 in SLC group and combination therapy group from day 7 to 9. In our opinion, this might be caused by the duration of treatment and progression of the tumor in vivo, which could be also inferred from other studies [6,17]. In support of this, we found the frequency of Tregs increased whereas the CD8^+^ T cells and CD4^+^ T cells simultaneously dropped in tumors in both the treated groups from day 7 to 9. These observations provided useful information on the time window for immunotherapies in HCC.

We observed that the combination therapy had the highest level of CD8^+^ T cells and modest level of CD4^+^ T cells. We considered that the reduced proportion of CD4^+^ T cells was due to depletion of CD4^+^ Tregs by anti-CD25 mAbs. As for the increased CD8^+^ T cells, we reasoned two possibilities: (1) Depletion of Tregs in the TME elicited a relatively suppression-free milieu, which facilitated the proliferation of CD8^+^ T cells [21,22]; (2) The residual Tregs were inactivated and reprogramed by anti-CD25 mAbs [23-25], which supported the recruitment and survival of CD8^+^ T cells. This change represented one of the anti-tumor effects.

In TME, we detected a higher level of IL-12 and IFN-γ (enhancing anti-tumor immunity) and lower level of TGF-β1 and IL-10 (suppressing anti-tumor immunity) in both treated groups. However, the levels of the four cytokines were not statistically significantly different between the SLC group and the combination therapy group. As we detected these cytokines on day 5, we were not sure whether these cytokines were significantly different at the other time points. More experiments are needed to exactly verify the cytokines profiles in TME during the tumor progression.

Unexpectedly, we found the frequency patterns of Tregs, CD8^+^ T cells and CD4^+^ T cells showed similar optimization in the lymph nodes, spleen and liver as that in TME, which manifested decreased Tregs and increased CD8^+^ T cells and CD4^+^ T cells. It has not been reported that SLC-based local treatments can lead to the systemic optimization of the immune cells in peripheral immune organs. It is not clear how this systemic optimization occurs. We supposed that the altered profiles of cytokines in TME, such as increased the IL-12 and IFN-γ and decreased TGF-β1 and IL-10, might exert some effects on peripheral organs. Indeed, some studies have already confirmed that TME contains many components that can affect the remote immune organs, e.g. the tumor-derived microvesicles and TLR4 ligands, which induce apoptosis of T cells [26,27]. Clinical studies also have found that HCC derived soluble factors simultaneously increased the number of Tregs and enhanced their suppressive function [28]; removing the tumors by surgery leads to systemically changed profiles of immune cells, such as decreased Tregs and granulocytes, and increased dendritic cells and CD4^+^ T cells [29,30].

The therapeutic effects of the combination therapy were assessed by both the growth and invasiveness of tumors. We found the growth of tumors was significantly reduced in the combination therapy and SLC group compared with the control group. Although the SLC treatment alone decreased the growth of tumors, the combined treatment manifested much more profound inhibition. This result confirmed our hypothesis that the combination therapy treatment would elicit improved efficacy, concordant with other studies combining SLC with different adjuvants [5,31]. MMP2 and MMP9 are important mediators for tumor progression and metastasis [32], and in our experiments we observed the levels of pro- and active-MMP2, MMP9 were the lowest in the combination therapy group among three groups. Therefore, these results demonstrated the efficacy of the combination therapy.

Interestingly, we noticed that the SLC group showed modest improvement compared with the control group and combination therapy group as indicated by the experiments in vivo and in vitro. These observations were consistent with previous studies from our lab and others which demonstrated the anti-tumor effects of SLC [3,7]. Importantly, although we did not found stronger anti-tumor effects of anti-CD25 treatment alone than that of SLC treatment alone in our preliminary experiments (data not shown), in the present study we found anti-CD25 mAbs treatment greatly boosted the anti-tumor effects of SLC, proving the efficacy of the combination therapy.

The DCs at the tumor sites are the key components for anti-tumor effects and a group of studies have confirmed the immature DCs profoundly counteract these benefits [33,34]. Intratumoral administration of SLC has been demonstrated to induce the maturation of DCs at tumor sites and this maturation constitutes one of the crucial anti-tumor effects elicited by the SLC-based treatments [6,16], which can partly explain our results. However, little is known about the detailed mechanisms of the SLC-CCR7 induced maturation. Our array data suggested that SCL-CCR7 axis resulted in up-regulation of genes in NF-κB pathway, which are important in the maturation of DCs [35,36]. Indeed, we found SLC effectively induced the maturation of BMDCs via NF-κB p65 in vitro. This finding was highly consistent with the recently published research describing CCR2 mediated maturation of DCs through the NF-κB pathway [37]. In addition, we also demonstrated that SLC and ELC, the two ligands for CCR7, had the same stimulation effects on BMDCs. These findings were very important for answering why these two ligands for CCR7 exhibit partially overlapping expression profiles, which was argued by Reinhold Fröster et al. [38]. Interestingly, we observed more potent SLC-induced phosphorylation of NF-κB p65 than ELC, and this might explain why SLC is the prior ligand for CCR7 in vivo [2]. Collectively, these results validated the new circuit underlying the maturation of DCs.

## Conclusions

Taken together, our results demonstrated that the intratumoral administration of SLC and anti-CD25 mAbs was an effective treatment for HCC in murine model. This combination therapy not only altered the profiles of Tregs, CD4^+^ T cells and CD8^+^ T cells but also influenced the cytokines (IL-12, IFN-γ, IL-10 and TGF-β1) at tumor sites. These changes constituted an anti-tumor environment in TME. Moreover, the combination therapy systemically optimized the profiles of Tregs, CD4^+^ T cells and CD8^+^ T cells in peripheral immune organs. The *in vitro* experiments confirmed that SLC induced the maturation of DCs through NF-κB p65, which was important for the SLC-based treatments. These results provided an appealing strategy in immunotherapy for HCC.

## Methods

### Animals and cell lines

C57BL/6 J (H-2b) female mice, 6–8 weeks of age, were purchased from the Chinese Academy of Science and housed at the Animal Maintenance Facility of Shanghai Medical College, Fudan University. All animal experiments were performed according to the regulations of Institutional Care of Experimental Animals Committee of Fudan University. Hepa 1–6, the murine hepatocellular carcinoma cell line (CRL-1830, ATCC, USA), was cultured in DMEM (Biowest, France) with 10% heat-inactivated FBS (GIBCO-BRL, USA), 0.1 mM nonessential amino acids, 1 μM sodium pyruvate, 2 mM L-glutamine, 100 μg/ml streptomycin, 100 units/ml penicillin, 50 μg/ml gentamicin, and 0.5 μg/ml fungizone.

### Establishment of murine models bearing HCC

The Hepa 1–6 tumor bearing mice were established as following: A total of 3 × 10^6^ Hepa 1–6 cells diluted in 200 μl of RPMI 1640 (Biowest) medium were injected subcutaneously into the right flank of the mice. One week after inoculation, the mice with visible tumor were examined for further experiments.

### Treatment of established HCC in mice

On day 8 after inoculation, tumor-bearing mice were randomly divided into 3 groups receiving different intratumoral injections: (1) control group: 50 μl rat IgG1 (2.5 μg/dose in saline diluent, eBioScience, USA); (2) SLC group: 50 μl recombinant murine SLC (0.5 μg/dose, PeproTech, USA); (3) combination therapy group: 50 μl recombinant SLC (0.5 μg/dose) and rat anti mouse CD25 monoclonal antibody (clone PC61.5, 2.5 μg/dose, eBioScience). All the injections were administered three times, once every other day. Tumor size and weight was monitored every other day for 9 days after the last injection. Tumor volume was calculated by the formula: *V* (in mm^3^) = 0.4(*ab*^*2*^), where *a* was the long diameter and *b* was the short diameter.

### IHC analysis

Tumor samples were embedded in OCT compound (Sakura, Japan) before being snap frozen in liquid nitrogen and stored at − 76°C until immunohistochemical procedures were performed. Serial 5-μm-thick cryostat sections were prepared and stained with antibodies against Foxp3, CCR7, CD4, CD8 (eBioScience).

### Western blot

Tumor tissues on day 1 to 9 post-treatment were homogenized with RIPA lysis buffer (Beyotime, China) and equal amounts of denatured proteins were used for immunoblotting with antibodies against CCR7, Foxp3 (eBioScience). BMDCs were pretreated with PDTC (100 μM, Sigma), the specific inhibitor of NF-κB activation, for 1 h and then stimulated with SLC (200 ng/ml) or ELC (200 ng/ml, PeproTech) for 1, 2, 4, 6, 12, 24 h. Cell lysates were immunoblotted with antibodies against CCR7, NF-κB p65, phosphorylated NF-κB p65, β-actin (Cell Signaling Technology) and Lamin B1 (Santa Cruz, USA). Band intensities were quantified using Band Leader software (Magnitec, Israel).

### Flow cytometry

Tumors, lymph nodes, spleens and livers were mechanically dissociated to produce a single cell suspension. Cells were then stained with antibodies against CD4, CD25, Foxp3, CD8 (eBioScience). BMDCs stimulated with SLC in the presence or absence of PDTC were stained with CCR7, CD80 and CD86 (eBioscience). Isotype-matched antibodies were used as controls. All the samples were acquired on a FACSCalibur (Becton Dickinson, USA) and analyzed with WinMDI 2.9 (USA).

### ELISA for cytokines

Tumor tissues were collected and homogenized on day 5 post-treatment and supernatants were used for evaluating cytokines (IL-12, IFN-γ, IL-10, and TGF-β1) using commercially available ELISA kits (eBioScience). Data was expressed at pg/mg tumor tissue.

### Gelatin zymography

Tumor tissues were homogenized on day 5 post-treatment and loaded onto zymographic sodium dodecyl sulfate gel containing gelatin (1 mg/ml, Sigma, Germany). Then the gels were incubated for 24 h at 37°C. The enzyme activity was visualized by staining the gel with Coomassie Blue R-250.

### Generation of BMDCs and SLC/ELC stimulation

Mouse BMDCs (bone-marrow-derived dendritic cells) were generated as previous described [3]. In brief, bone marrow cells were cultured in RPMI 1640 containing 10% FBS with GM-CSF, IL-4 and tumor necrosis factor-α (TNF-α). Non-adherent cells were harvested and sorted by anti-CD11c microbeads (Miltenyi, Germany). Analysis by flow cytometry revealed that the purity of the BMDCs was consistently more than 90%, and the phenotype was characterized as MHC II^high^ CD86^low^. Aliquots of 2 × 10^6^ cells were incubated in RPMI 1640 with 10% FBS and HEPES buffer for 30 min, and were then stimulated with SLC or ELC.

### G protein-coupled receptors signaling PathwayFinder gene array

Total RNAs were purified from BMDCs stimulated with SLC for 1 or 12 hours by Trizol reagent (Invitrogen, USA). RNAs were arrayed by the G Protein-coupled Receptors Signaling PathwayFinder Gene Array (SuperArray Bioscience Corporation, USA). Differentially expressed genes were identified according to GeneChip® Expression Analysis-Data Analysis Fundamentals. The arrays were repeated 3 times. Data-mining tools used in this study included PubMatrix (http://pubmatrix.grc.nia.nih.gov), Gene Map Annotator and Pathway Profiler 2.0 (http://www.GenMapp.org).

### Statistical analysis

For comparisons of the various treatment groups, ANOVA was performed with the Bonferroni correction. All statistical analyses were performed using the SPSS statistical software package (SPSS 20.0 for Windows, USA). Differences were considered statistically significant with * *P* < 0.05 and highly significant with ** *P* < 0.01.

## Abbreviations

Tregs: Regulatory T cells; DCs: Dendritic cells; SLC: Secondary lymphoid tissue chemokine; ELC: EBI1 ligand chemokine; BMDCs: Bone-marrow-derived dendritic cells; HCC: Hepatocellular carcinoma; TME: Tumor microenvironment; IHC: Immunohistochemistry; FACs: Flow cytometry.

## Competing interests

The authors declare that they have no competing interests.

## Authors’ contributions

LC, SZ and JQ performed the experiments, interpreted the findings and prepared the manuscript. HH and HM assisted with the FACs and Western blot. BL performed the statistical analysis. XW and JM assisted in animals maintenance. SY and CZ critically read the manuscript. GZ provided practical advice on experiments. CL conceived of the study, participated in the design, and assisted with data interpretation and manuscript writing. All authors read and approved the final manuscript.

## References

[B1] Forster R Davalos-Misslitz AC Rot A CCR7 and its ligands: balancing immunity and tolerance Nat Rev Immunol 2008 12 362 371 10.1038/nri2297 18379575

[B2] Britschgi MR Favre S Luther SA CCL21 is sufficient to mediate DC migration, maturation and function in the absence of CCL19 Eur J Immunol 2010 12 1266 1271 10.1002/eji.200939921 20201039

[B3] Liang CM Ye SL Zhong CP Zheng N Bian W Sun RX Chen J Li RL Zhou S Liu YK More than chemotaxis: a new anti-tumor DC vaccine modified by rAAV2-SLC Mol Immunol 2007 12 3797 3804 10.1016/j.molimm.2007.03.026 17521735

[B4] Riedl K Baratelli F Batra RK Yang SC Luo J Escuadro B Figlin R Strieter R Sharma S Dubinett S Overexpression of CCL-21/secondary lymphoid tissue chemokine in human dendritic cells augments chemotactic activities for lymphocytes and antigen presenting cells Mol Cancer 2003 12 35 10.1186/1476-4598-2-35 14613584 PMC270078

[B5] Nguyen-Hoai T Baldenhofer G Sayed Ahmed MS Pham-Duc M Vu MD Lipp M Dorken B Pezzutto A Westermann J CCL21 (SLC) improves tumor protection by a DNA vaccine in a Her2/neu mouse tumor model Cancer Gene Ther 2012 12 69 76 10.1038/cgt.2011.69 21997231

[B6] Turnquist HR Lin X Ashour AE Hollingsworth MA Singh RK Talmadge JE Solheim JC CCL21 induces extensive intratumoral immune cell infiltration and specific anti-tumor cellular immunity Int J Oncol 2007 12 631 639 17273764

[B7] Liang CM Zhong CP Sun RX Liu BB Huang C Qin J Zhou S Shan J Liu YK Ye SL Local expression of secondary lymphoid tissue chemokine delivered by adeno-associated virus within the tumor bed stimulates strong anti-liver tumor immunity J Virol 2007 12 9502 9511 10.1128/JVI.00208-07 17567706 PMC1951415

[B8] Rudensky AY Regulatory T cells and Foxp3 Immunol Rev 2011 12 260 268 10.1111/j.1600-065X.2011.01018.x 21488902 PMC3077798

[B9] Josefowicz SZ Lu LF Rudensky AY Regulatory T cells: mechanisms of differentiation and function Annu Rev Immunol 2012 12 531 564 10.1146/annurev.immunol.25.022106.141623 22224781 PMC6066374

[B10] Gao Q Qiu SJ Fan J Zhou J Wang XY Xiao YS Xu Y Li YW Tang ZY Intratumoral balance of regulatory and cytotoxic T cells is associated with prognosis of hepatocellular carcinoma after resection J Clin Oncol 2007 12 2586 2593 10.1200/JCO.2006.09.4565 17577038

[B11] Ormandy LA Hillemann T Wedemeyer H Manns MP Greten TF Korangy F Increased populations of regulatory T cells in peripheral blood of patients with hepatocellular carcinoma Cancer Res 2005 12 2457 2464 10.1158/0008-5472.CAN-04-3232 15781662

[B12] Shen X Li N Li H Zhang T Wang F Li Q Increased prevalence of regulatory T cells in the tumor microenvironment and its correlation with TNM stage of hepatocellular carcinoma J Cancer Res Clin Oncol 2010 12 1745 1754 10.1007/s00432-010-0833-8 20221638 PMC11827820

[B13] Setiady YY Coccia JA Park PU In vivo depletion of CD4 + FOXP3+ Treg cells by the PC61 anti-CD25 monoclonal antibody is mediated by FcgammaRIII + phagocytes Eur J Immunol 2010 12 780 786 10.1002/eji.200939613 20039297

[B14] Morse MA Hobeika AC Osada T Serra D Niedzwiecki D Lyerly HK Clay TM Depletion of human regulatory T cells specifically enhances antigen-specific immune responses to cancer vaccines Blood 2008 12 610 618 10.1182/blood-2008-01-135319 18519811 PMC2481547

[B15] Byrne WL Mills KH Lederer JA O’Sullivan GC Targeting regulatory T cells in cancer Cancer Res 2011 12 6915 6920 10.1158/0008-5472.CAN-11-1156 22068034 PMC4287207

[B16] Oh SM Oh K Lee DS Intratumoral administration of secondary lymphoid chemokine and unmethylated cytosine-phosphorothioate-guanine oligodeoxynucleotide synergistically inhibits tumor growth in vivo J Korean Med Sci 2011 12 1270 1276 10.3346/jkms.2011.26.10.1270 22022177 PMC3192336

[B17] Li X Kostareli E Suffner J Garbi N Hammerling GJ Efficient Treg depletion induces T-cell infiltration and rejection of large tumors Eur J Immunol 2010 12 3325 3335 10.1002/eji.201041093 21072887

[B18] Morita R Hirohashi Y Sato N Depletion of Tregs in vivo: a promising approach to enhance antitumor immunity without autoimmunity Immunotherapy 2012 12 1103 1105 10.2217/imt.12.116 23194360

[B19] Ladoire S Arnould L Mignot G Coudert B Rebe C Chalmin F Vincent J Bruchard M Chauffert B Martin F Presence of Foxp3 expression in tumor cells predicts better survival in HER2-overexpressing breast cancer patients treated with neoadjuvant chemotherapy Breast Cancer Res Treat 2011 12 65 72 10.1007/s10549-010-0831-1 20229175

[B20] Lu H FOXP3 expression and prognosis: role of both the tumor and T cells J Clin Oncol 2009 12 1735 1736 10.1200/JCO.2008.20.0675 19255314

[B21] Zhang HH Mei MH Fei R Liao WJ Wang XY Qin LL Wang JH Wei L Chen HS Regulatory T cell depletion enhances tumor specific CD8 T-cell responses, elicited by tumor antigen NY-ESO-1b in hepatocellular carcinoma patients, in vitro Int J Oncol 2010 12 841 848 20198327 10.3892/ijo_00000561

[B22] Fu J Xu D Liu Z Shi M Zhao P Fu B Zhang Z Yang H Zhang H Zhou C Increased regulatory T cells correlate with CD8 T-cell impairment and poor survival in hepatocellular carcinoma patients Gastroenterology 2007 12 2328 2339 10.1053/j.gastro.2007.03.102 17570208

[B23] Rech AJ Mick R Martin S Recio A Aqui NA Powell DJ Jr Colligon TA Trosko JA Leinbach LI Pletcher CH CD25 blockade depletes and selectively reprograms regulatory T cells in concert with immunotherapy in cancer patients Sci Transl Med 2012 12 134 ra162 10.1126/scitranslmed.3003330 PMC4425934 22593175

[B24] Teng MW Ngiow SF von Scheidt B McLaughlin N Sparwasser T Smyth MJ Conditional regulatory T-cell depletion releases adaptive immunity preventing carcinogenesis and suppressing established tumor growth Cancer Res 2010 12 7800 7809 10.1158/0008-5472.CAN-10-1681 20924111

[B25] Zhou X Bailey-Bucktrout S Jeker LT Bluestone JA Plasticity of CD4(+) FoxP3(+) T cells Curr Opin Immunol 2009 12 281 285 10.1016/j.coi.2009.05.007 19500966 PMC2733784

[B26] Wieckowski EU Visus C Szajnik M Szczepanski MJ Storkus WJ Whiteside TL Tumor-derived microvesicles promote regulatory T cell expansion and induce apoptosis in tumor-reactive activated CD8+ T lymphocytes J Immunol 2009 12 3720 3730 10.4049/jimmunol.0900970 19692638 PMC3721354

[B27] Liu YY Sun LC Wei JJ Li D Yuan Y Yan B Liang ZH Zhu HF Xu Y Li B Tumor cell-released TLR4 ligands stimulate Gr-1 + CD11b + F4/80+ cells to induce apoptosis of activated T cells J Immunol 2010 12 2773 2782 10.4049/jimmunol.1000772 20675592

[B28] Cao M Cabrera R Xu Y Firpi R Zhu H Liu C Nelson DR Hepatocellular carcinoma cell supernatants increase expansion and function of CD4(+)CD25(+) regulatory T cells Lab Invest 2007 12 582 590 17372588 10.1038/labinvest.3700540

[B29] Kaufman HL Kim DW DeRaffele G Mitcham J Coffin RS Kim-Schulze S Local and distant immunity induced by intralesional vaccination with an oncolytic herpes virus encoding GM-CSF in patients with stage IIIc and IV melanoma Ann Surg Oncol 2010 12 718 730 10.1245/s10434-009-0809-6 19915919

[B30] Hase S Weinitschke K Fischer K Fornara P Hoda R Unverzagt S Seliger B Riemann D Monitoring peri-operative immune suppression in renal cancer patients Oncol Rep 2011 12 1455 1464 21369703 10.3892/or.2011.1199

[B31] Thanarajasingam U Sanz L Diaz R Qiao J Sanchez-Perez L Kottke T Thompson J Chester J Vile RG Delivery of CCL21 to metastatic disease improves the efficacy of adoptive T-cell therapy Cancer Res 2007 12 300 308 10.1158/0008-5472.CAN-06-1017 17210711

[B32] Kessenbrock K Plaks V Werb Z Matrix metalloproteinases: regulators of the tumor microenvironment Cell 2010 12 52 67 10.1016/j.cell.2010.03.015 20371345 PMC2862057

[B33] Fainaru O Almog N Yung CW Nakai K Montoya-Zavala M Abdollahi A D’Amato R Ingber DE Tumor growth and angiogenesis are dependent on the presence of immature dendritic cells FASEB J 2010 12 1411 1418 10.1096/fj.09-147025 20008545 PMC2879945

[B34] Ghiringhelli F Puig PE Roux S Parcellier A Schmitt E Solary E Kroemer G Martin F Chauffert B Zitvogel L Tumor cells convert immature myeloid dendritic cells into TGF-beta-secreting cells inducing CD4 + CD25+ regulatory T cell proliferation J Exp Med 2005 12 919 929 10.1084/jem.20050463 16186184 PMC2213166

[B35] Dissanayake D Hall H Berg-Brown N Elford AR Hamilton SR Murakami K Deluca LS Gommerman JL Ohashi PS Nuclear factor-kappaB1 controls the functional maturation of dendritic cells and prevents the activation of autoreactive T cells Nat Med 2011 12 1663 1667 10.1038/nm.2556 22081022

[B36] Jung MY Kim HS Hong HJ Youn BS Kim TS Adiponectin induces dendritic cell activation via PLCgamma/JNK/NF-kappaB pathways, leading to Th1 and Th17 polarization J Immunol 2012 12 2592 2601 10.4049/jimmunol.1102588 22345647

[B37] Jimenez F Quinones MP Martinez HG Estrada CA Clark K Garavito E Ibarra J Melby PC Ahuja SS CCR2 plays a critical role in dendritic cell maturation: possible role of CCL2 and NF-kappa B J Immunol 2010 12 5571 5581 10.4049/jimmunol.0803494 20404272 PMC2929965

[B38] Worbs T Forster R A key role for CCR7 in establishing central and peripheral tolerance Trends Immunol 2007 12 274 280 10.1016/j.it.2007.04.002 17462953

